# Uncovering success stories: how to resuscitate in situ simulation initiatives in Canadian emergency departments

**DOI:** 10.1186/s41077-025-00376-w

**Published:** 2025-09-29

**Authors:** Laurence Baril, Kyla Caners, Melanie Walker, Damon Dagnone, Tim Chaplin, Éliane Raymond-Dufresne, Jared Baylis, Eve Purdy, Samantha Britton, Christine Cash

**Affiliations:** 1 Département de Médecine Familiale et Médecine d’urgence, Faculté de Médecine,, Université Laval, QC Québec, Canada; 2 Département de Médecine d’urgence, CHU de Québec, Québec, QC Canada; 3 Department of Emergency Medicine, Queen’s University, Kingston, Canada; 4 Department of Emergency Medicine, University of British Columbia, Vancouver, Canada; 5 Emergency Department, Gold Coast University Hospital, Gold Coast, Australia; 6 Faculty of Health Sciences & Medicine, Bond University, Gold Coast, Australia

**Keywords:** In Situ simulation, Translational simulation, Positive deviants, Simulation programs, Leadership in simulation

## Abstract

In situ simulation (ISS) has long been recognized as a powerful tool for identifying latent safety threats, enhancing teamwork, and ultimately improving patient safety in Emergency Departments (EDs). However, the challenges of operationalizing ISS training in the current clinical environment in Canadian EDs have become increasingly evident. While many EDs face hurdles in implementing ISS, some teams have proven resilient and successful in their ISS endeavors. This study aims to determine which factors are associated with the successful maintenance of ISS programs within Canadian EDs. Using a positive deviance approach, we conducted a qualitative study of ED teams engaged in ISS projects, using interviews as a data collection tool. We recruited 14 healthcare providers who had participated in successful ISS initiatives in Canadian EDs. Participants highlighted the importance of engaging interprofessional stakeholders, flexibility from the simulation team, and buy-in from participants and colleagues as key factors contributing to the success of ISS programs. Challenges identified included lack of buy-in, space constraints, high patient volume and acuity, and staff shortages. Strategies for managing these challenges included scheduling simulations during less busy times and having alternative spaces for simulations. ISS was found to have a significant impact on patient safety, improving teamwork, crisis resource management, and overall patient care. These findings provide valuable insights for EDs looking to start or improve their ISS programs, emphasizing the importance of collaboration and adaptability in overcoming challenges to ensure the success of ISS initiatives.

## Background

Translational simulation has been recognized as a powerful tool to enhance the performance of healthcare teams and systems [1–5] yet, the uptake of this method for team and system improvement has been variable across Canadian emergency departments. In situ simulation (ISS)—simulation conducted within real clinical spaces with authentic clinical teams—is one common modality within translational simulation that has unique opportunities but also unique barriers to implementation, including competing patient demands, environmental challenges, and scheduling conflicts [6]. Despite near-universal environmental pressures faced by Canadian emergency departments, there are some programs that successfully overcome barriers to conducting ISS. Understanding the success of these outlier programs will offer critical insights for those hoping to start or improve their own translational simulation programs.

Canadian EDs could be a poster child for environmental pressures that prevent the implementation of ISS. They face numerous challenges, including access-block, overcrowding, and lack of human resources [7]. Patients are frequently assessed in hallways, and departments usually operate at well over 100% capacity [8]. Despite this overwhelming environmental burden, over the last few years, we have become aware of a handful of groups that have operationalized ISS within these harsh conditions. We wanted to know what made these outliers successful.

Positive deviance is a method that focuses on identifying and learning from individuals or groups who have achieved better outcomes or performance, despite facing similar challenges and constraints as their peers [9, 10]. A deep exploration of their success can elicit unique practices, strategies, and behaviors that contributed to their success with the expectation that others can learn from their experience [9].

Using a positive deviance approach, the objective of this study was to identify key features, strategies, and adaptations of resilient ISS programs. A secondary objective was to offer practical solutions to common challenges in implementing ISS, with the aim of supporting other medical teams.

## Methods

From a constructivist paradigm and using the guiding framework of positive deviance, we performed a qualitative interview study with ISS leaders.

### Study population

We recruited simulation leaders from EDs across Canada through a combination of self-identification and peer nomination. An email was sent to an existing list of Canadian simulation education researchers (Emergency Medicine Simulation Education Researchers of Canada, EM-SERC) searching for self-identified or peer-nominated examples of positive deviants. In this context, positive deviants were defined as teams that are accomplishing any form of “successful” in situ simulation activity within their ED. The term successful was meaningfully left to interpretation since we felt that defining success was likely context dependent and an outcome best assessed by those who were familiar with the objectives of their specific programs. No strict criteria in terms of frequency, duration, or structure were imposed. EM-SERC was used as a starting point as this group includes simulation educators representing every Royal College of Physicians and Surgeons in Emergency Medicine training program across the country. Study participants were included if they participated in any sort of ISS program in a Canadian ED, spoke English or French, and were willing to share their experience with the research team. No willing participant was excluded in the process.

The objective was to recruit until reaching data sufficiency of identified themes, as described in the qualitative research literature [11, 12].

### Data collection

One member of each of these identified teams underwent a semi-structured interview with the principal investigator. The interviews aimed to uncover the strategies and innovations that have enabled these teams to surmount the barriers that often impede ISS in the ED. An interview guide was developed based on the “Input-Process-Output framework for translational simulation” (1) and was pilot-tested with a simulation expert. The guide was refined based on feedback before being used in the study. The interview template can be found in Appendix 1.

Verbal consent was obtained and recorded prior to the interviews. All interviews were conducted through the online video-conferencing platform Zoom. They were recorded and transcribed verbatim by the principal investigator [13]. The French interviews were translated to English by the primary investigator to facilitate the thematic analysis.

### Data analysis

**Fig. 1:**
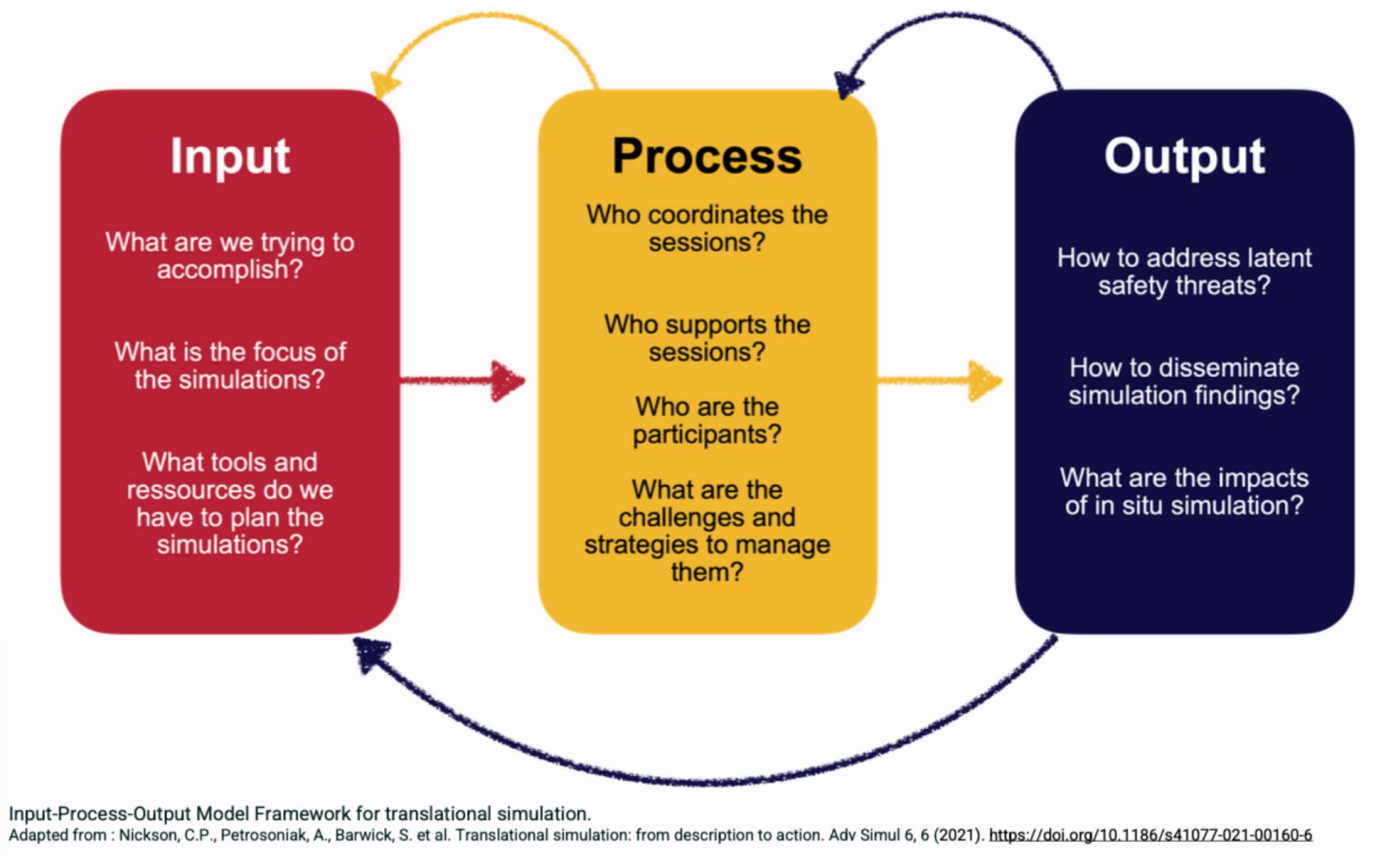
Input-Process-Output Framework for translational simulation

An inductive thematic analysis, sensitized by the input-process-output model, was then performed on all interview transcripts by two independent reviewers (LB, CC) using the NVivo12 software (NVivo Pro Qualitative Analysis Software. QSR International; 2021). The input-process-output model has been applied to ISS by previous authors [6] and dissects the planning, realization, and application of translational simulation (Fig. 1).

The two reviewers regularly met to assure coherence through the analysis of data. Discrepancies were resolved through discussion until consensus was reached. This study received ethical approval from Queen’s University Health Sciences Research Ethics (TRAQ # 6,037,884). Informed consent was obtained from all participants.

## Results

### Characteristics of study participants

Between March and July 2023, we recruited a convenience sample of 14 healthcare providers who had actively participated in successful ISS initiatives within Canadian EDs. This group comprised ten emergency physicians and four nurse educators. Notably, the majority [12] of our participants were affiliated with academic hospitals, while two were based in community centers. Participants could identify with multiple site types; therefore, numbers and percentages add up to more than *N* = 14 and 100%. Our sample represented a diverse geographic spectrum, with participants coming from six different provinces: Quebec (4), Ontario (3), British Columbia (3), Alberta (2), Saskatchewan (1), and Nova Scotia (1) (Table 1). Interviews were conducted in French with the Quebec-based participants to ensure a seamless and authentic conversation in their first language.

**Table 1:** Demographic data

Type of site	*N*	%
Academic	12	86%
Community	4	29%
Rural	1	7%
Profession		
ED physician	10	71%
Nurse	4	29%
Province		
Alberta	3	21%
British-Columbia	3	21%
Nova Scotia	1	7%
Ontario	4	29%
Quebec	4	29%
Saskatchewan	1	7%

Ten participants described simulations happening monthly, two every other week, one annually, one weekly, and one on an irregular basis. All simulation activities were interprofessional, including at least nursing and medical staff, and most also involved respiratory therapists. All interviewed participants were practicing professionals who had completed their formal training.

## Thematic analysis

### Definition of success

Firstly, we were interested in understanding how facilitators defined success of their programs. The main themes identified were *consistency* and *sustainability*. Further contributors to a sense of success were the feeling that participants were improving the quality of patient care and creating a workplace culture in which ISS was welcomed and valued.

As a participant working in an academic center described, “Success means simulation happens, for one, because that's a huge challenge. I think that it's been from a large part engagement from key stakeholders. I think there's an organizational culture that's developing that supportive of it. And now starting to see the benefits of it.” (Participant 7).

### Input-process-output

The Input-Process-Output model (1) for translation simulation informed a more granular understanding of the contributors to success.

### Input

The input aspect of the translational simulation model described above refers to the objectives, planning, and resources involved in orchestrating the simulations.

### Clarity of purpose

The facilitators interviewed had clear objectives for their ISS programs. Targeted objectives of successful ED ISS were generally described by participants in two broad categories: (1) *to improve teamwork* and (2) *to evaluate and improve processes and protocols*. Some facilitators described one of these core objectives; sometimes, they described both. Even within a program, there was recognized variation from simulation to simulation. Secondary objectives included wanting to familiarize staff with the working environment and teaching medical learners and nursing staff.

#### Leadership

There was professional variation in the leads for simulation planning, with some sites having nursing educators and others having physicians as the primary leads. In most cases, however, the preparatory work was shared between both nurse and physician leads. Furthermore, all respondents described the support from nurse managers and departmental leaders as essential to a sustainable simulation program.

#### Funding

There was wide variation in funding within the positive deviants. Four programs had a designated funding source allocated for ISS—two worked with a fund created by emergency physicians to support the ISS program, and two others operated on grants and hospital financial support. The role of a simulation lead was a funded position in these four programs. In half of the programs, physicians were allocated Continuing Medical Education (CME) credits, which were recognized as an important incentive for physician participation. One third of teams did not have a simulation technician available to help support the ISS program. In these cases, members of the clinical team took on that role.

### Process

The process aspect of the model addressed the coordination, support, and participation in the simulation sessions.

#### Collaboration

Simulations were described as successful when they were interprofessional and, in the case of four respondents, designed in collaboration with other departments. Success was not dependent on the profession of the primary lead, but in all cases, collaboration between different professional groups was necessary for success.

#### Staffing

In most programs, participants for the ISS sessions were recruited in advance as additional staff that would come in specifically for the simulation. This minimized the impact on simultaneous clinical demands.

#### Flexibility

The ability to be flexible and to adapt to the context of the department was highlighted as a key component of success. Many facilitators described that the topics of the simulation they designed were responsive to the needs of the department. For example, some simulation topics were inspired by actual cases or by perceived team needs. Other aspects of simulation design, including modality and duration, were carefully considered. As an example, a participant mentioned using shorter cases, targeted to specific objectives, to take less time in the department. Being able to “grab and go”, or in other words leave the resuscitation area if needed, was important to participants.

#### Environment and safety

Conducting ISS in an overcrowded ED was mentioned by all as being a perpetual challenge. To manage this, teams kept a regular schedule for simulations and were careful to select times when EDs were typically less busy. Some also mentioned having an alternative space as a backup if the resuscitation room was occupied by a real patient.

Most participants had ‘no-go’ criteria, described as considerations under which ISS should be cancelled [14]. Three respondents used a formal checklist prior to each session to evaluate the safety of performing the simulation in the ED. Many recalled having to cancel the simulation if the acuity or volume of patients in the department was too high, and all reported that this was a collaborative, interprofessional decision between charge nurses, physicians on shift, and the simulation team.

#### Psychological safety

Many programs described specific strategies to enhance psychological safety such as pre-briefing and almost all indicated they shared the cases, or some elements of them, in advance to participants. One interviewee outlined the need to reshape participants’ understanding of the purpose of ISS—away from testing and judgment they may have experienced in training.

“we try to in the prebrief to talk about psychological safety […]. And every time I say the same mantra: we're not here to test you, we're not here to judge you, we're here to test our system.” (Participant 4).

### Debriefing

Finally, the quality of the debrief was underlined multiple times as an important key to success and buy-in to ISS. Who ran the debriefing was variable through the teams, including physicians, nursing staff, and other simulation experts. The debrief habitually focused on team dynamics and identified threats to patient safety.

### Output

Our participants reflected on how the impacts of ISS were obvious at the organizational, team, and individual levels.

#### Departmental

The departmental effects of ISS predominantly involved processes and protocols, which were described as safer and more fluid after having practiced them in the real clinical setting. Nearly all the programs used simulation to identify potential threats to safety but had various mechanisms for reporting and actioning these risks. Most often, responsibility was taken on by the nurse educator and reported to appropriate personnel for follow-up. Some participants also used the hospital local quality improvement system to do so. A successful strategy described was to include administrators in the post-simulation report to facilitate the transmission of information. Many mentioned the importance of sharing simulation findings with the simulation participants, improving buy-in and making tangible the impacts of in situ simulation. Collecting feedback from participants was also deemed important to improve collaboration on future simulation activities.

#### Team

All facilitators felt that there were important, but “intangible” outcomes related to interprofessional collaboration. Simulation was noted to increase communication and psychological safety with the team and to flatten hierarchy, as well as to foster a culture of development among teams.

“The impacts are broad and predominantly positive. And then there's just probably the intangibles like training together, and then it just helps build teamwork and team familiarity” (Participant 1).

“Because by getting to practice in these situations, and [the team] feeling more empowered to speak up, I think it's nice, from a system perspective to break down some of the kind of conventional hierarchical views” (Participant 3).

#### Individual

While individual level learning outcomes were not the objective for most ISS programs, many facilitators described ISS as greatly improving people’s confidence and comfort in taking care of critically ill patients. Many mentioned an improvement in medical knowledge through simulation, even if it wasn’t the primary objective of the activity. Facilitators felt that ISS was of benefit to emergency providers of all levels of training, from new recruits to those with the most experience.

### Challenges in in situ simulation

Despite successes, all facilitators faced very real challenges. The most common challenges encountered were lack of buy-in from colleagues, lack of space, ED overcrowding, and staff shortages, especially nursing staff. Lack of access to simulation technicians, unstable leadership, and burnout were also discussed as barriers to ISS programs. Overcoming these barriers largely relied on factors outlined above, including careful planning, alignment with departmental priorities, flexibility in delivery, commitment to cultivating psychological safety, and demonstration of impact.

What facilitators did not describe directly, but we could sense throughout the interviews, was that success also relied on extraordinary levels of personal commitment from this group—with many volunteering to coordinate sessions on their days off, using their own social capital to recruit participants, and sacrificing personal time to commit to ongoing learning about simulation and improvement techniques.

## Discussion

### Summary of findings

The findings from this study shed light on several key aspects of successfully implementing and conducting ISS programs in the ED. Participants emphasized the importance of defining success in ISS as not only achieving consistency and sustainability in conducting simulations, but also as fostering a workplace culture that values ISS itself. The challenges overcome by our facilitators were similar to those documented by other Canadian emergency providers in the EM:POWER Task Force Report (2024). But more interestingly, the spiral of successful ISS fostering a workplace culture that supports more ISS is well supported by Eller et al.’s description of three successful ISS programs in varied healthcare contexts [15]. So, what can we learn from the spiral of success in these Canadian EDs?

Central to the positive deviance approach is to identify strategies that might help others achieve similar outcomes [9]. As such, in this discussion we will outline recommendations for the input, process, and output for ISS in emergency departments based on our findings and in the context of other relevant literature. It is likely that these principles identified in the harsh environment of Canadian EDs will apply in other overburdened contexts. These recommendations, and accompanying literature, can be found in Table 2.

**Table 2:** Recommendations for ED ISS

Input	
	Recommendation
Clarity of Purpose	• Identify objectives (organizational and/or team) for the simulation program and each simulation activity • Match appropriate simulation approaches to the objectives (ISS may not be the most appropriate) given the significant practical and environmental challenges • Build time to reflect on whether simulation activities are meeting objectives and mission
Leadership	• Obtain support of medical and nursing leadership • Encourage leadership development in an interprofessional and collaborative perspective
Funding	• Within your context, identify resources (human and financial) to make simulation sustainable • Work with institutional simulation services and resources when available • Seek funding to enhance sustainability of the simulation program • If possible, obtain continuing medical/nursing education credits for participants
Process	
Collaboration	• Include medical and nursing staff in the design, delivery, and debriefing of simulation • If relevant to your simulation objectives, include other groups (interdepartmental, intradepartmental) in the design, delivery, and debriefing of simulations
Staffing	• The staff participating in ISS should be supernumerary to staff providing care to patients
Flexibility	• Adapt to the needs of the department in simulation design and delivery (ex: timing of simulations) • Have a backup plan for simulation delivery (ex: alternate location, use of different modality (i.e. visually enhanced mental simulation) (16) • Be ready to stop the simulation if resources become limited for patient care
Environment and Safety	• Develop a collaborative process for deciding whether to proceed with or cancel a simulation • Review or develop a simulation safety plan (strict or flexible no-go criteria)
Psychological Safety	• Recognize that departmental culture, and participants’ past experiences with simulation will significantly impact their experience with ISS • Engage in a pre-briefing for every simulation activity this time can be situationally adapted but in general should be used to: clarify objectives, attend to logistics, set expectations, highlight safety considerations, and foster respect • Recognize that participants’ experience in simulation will impact their work in the ED and their attitudes towards the next simulation activity
Debriefing	• Adapt your debriefing strategy to the objectives of the simulation • Commit to a focus on team and organizational learning
Output	
Organizational	• Consider collaborating with existing quality and safety mechanisms to develop reporting mechanisms • Develop a simulation reporting system that provides meaningful feedback to appropriate departmental leadership and participants • Communicate early wins widely
Team	• Reflect on the impact of simulation on team familiarity, trust, and affect • Role model a way for teams to reflect on their own real-world performance
Individual	• Though not a primary objective, participants often want to discuss individual performance. Consider creating time and space for participants to speak with each other after the formal debrief for these individually focused learning conversations

Our findings are coherent with previous research. The barriers identified in our study, including ED overcrowding and lack of buy-in, are consistent with findings from Jee M et al. (2023), who noted similar constraints in simulation programs globally. Their emphasis on the role of interdisciplinary collaboration and tailored solutions complements our recommendations for overcoming these challenges in Canadian EDs. The importance of strong leadership and support from administrative instances is proposed as a key element of successful ISS programs in the ED [17].

The input-process-output [18] model is cyclical, not linear. Successful programs were involved in a constant process of reflection, adaptation, and evolution. They were sensitive to the needs of the department at a programmatic and individual session level. The IPO model, and our findings, also invite those starting simulation programs to reflect carefully on clarity of purpose. While we explored ISS, it is possible that other modalities (with fewer challenges) may be better matched to objectives for overall programs or individual sessions. The questions that we used to guide our interviews, as presented in Fig. 1, can also be used to facilitate ongoing reflection for simulation leaders.

### Strengths and limitations

This study had limitations. The sample size was small but provided valuable insights and sufficient data to answer our questions about what contributes to success. It is possible that other strategies, not identified by our sample, exist; we should continue to interrogate outliers to identify other valuable lessons. The snowball recruitment method could cause a sampling bias; however, participants from different practice settings (academic, community, and rural) were included. The reliance on self-reported data could cause a social desirability bias and means that our interpretation is through the lens of facilitators, rather than participants or administrators. While this is a limitation, we feel that they are well positioned to reflect on the challenges and realities of program implementation. Additionally, the study focused exclusively on Canadian EDs, limiting the generalizability of the findings to other healthcare settings, which is relevant as it relates specifically to funding and staffing. Globally, many EDs face similar constraints, but other healthcare contexts will likely have challenges unique to their environments and systems. Finally, despite wanting to have a multiprofessional cohort, we did not have any respiratory therapists among our participants. Their participation in the study could have offered a different perspective and revealed challenges and solutions specific to this profession.

This study used a novel method, the positive deviance approach, to answer the research question. It aimed to provide the readers with realistic ideas and strategies to establish ISS in their own department based on concrete examples of successful simulation programs. We included both French and English speaking participants and conducted the interviews in participants’ preferred language; therefore, not limiting their ability to share information with the primary investigator. The qualitative thematic analysis helped us gain a deeper understanding of the elements supporting the success of ISS in the ED.

### Future directions

Future research in the field of ISS within EDs could explore the impact of ISS programs on patient outcomes, team performance, and organizational culture over the long term, providing valuable insights into their lasting effects and potential for continuous improvement. Additionally, further investigation into the optimal frequency, duration, and structure of ISS sessions could help refine ISS programs to maximize their effectiveness and feasibility within busy clinical environments. It is likely that, however, all of these factors are context dependent. It would be useful to generate a better understanding of when ISS is preferred to other simulation modalities with fewer practical barriers (i.e., visually enhanced mental simulation [16, 19]. Moreover, exploring the role of emerging technologies, such as virtual reality and artificial intelligence (AI), in enhancing ISS training and integration into ED workflows could offer new avenues for improving patient safety and healthcare provider performance, as described in the literature [20–23]. Given our unique observation of significant personal sacrifice and dedication of ISS leaders, it may also be worth understanding how personality, motivation, social capital, and charisma of ISS leaders impact success.

## Conclusion

This study provides valuable insights into the strategies and practices that contribute to the successful implementation of ISS programs in Canadian EDs. Despite the numerous challenges faced by Canadian EDs, including access block, overcrowding, and staff shortages, some teams have been able to overcome these barriers and conduct successful ISS programs. Key factors contributing to their success include engagement of interprofessional stakeholders, flexibility from the simulation team, and buy-in from participants and colleagues. These findings can serve as a guide for other EDs looking to start or improve their ISS programs. Overall, ISS has the potential to significantly improve patient care, teamwork, and safety in EDs, and efforts should be made to continue to enhance and expand these programs.

## Data Availability

No datasets were generated or analysed during the current study.

## Abbreviations

CME: Continuing Medical Education
ED: Emergency Departments
EM-SERC: Emergency Medicine Simulation Education Researchers of Canada
ISS: In Situ Simulation
